# Evaluation of Mechanical and Tribological Properties of Corn Cob-Reinforced Epoxy-Based Composites—Theoretical and Experimental Study

**DOI:** 10.3390/polym13244407

**Published:** 2021-12-14

**Authors:** Ahmed Fouly, Hany S. Abdo, Asiful H. Seikh, Khalid Alluhydan, Hend I. Alkhammash, Ibrahim A. Alnaser, Mohamed S. Abdo

**Affiliations:** 1 Mechanical Engineering Department, King Saud University, P.O. Box 800, Riyadh 11421, Saudi Arabia; kalluhydan@KSU.EDU.SA (K.A.); ianaser@KSU.EDU.SA (I.A.A.); 2 Department of Production Engineering and Mechanical Design, Faculty of Engineering, Minia University, Minia 61519, Egypt; 3 Center of Excellence for Research in Engineering Materials (CEREM), King Saud University, P.O. Box 800, Riyadh 11421, Saudi Arabia; habdo@ksu.edu.sa (H.S.A.); aseikh@ksu.edu.sa (A.H.S.); 4 Mechanical Design and Materials Department, Faculty of Energy Engineering, Aswan University, Aswan 81521, Egypt; 5 Department of Electrical Engineering, College of Engineering, Taif University, P.O. Box 11099, Taif 21944, Saudi Arabia; Khamash.h@tu.edu.sa; 6 Biomedical Engineering Department, Faculty of Engineering, Minia University, Minia 61519, Egypt; Bioengmsa@yahoo.com

**Keywords:** epoxy composite, green composite, corn cob

## Abstract

Epoxy is considered to be the most popular polymer and is widely used in various engineering applications. However, environmental considerations require natural materials-based epoxy. This necessity results in further utilization of natural materials as a natural reinforcement for different types of composites. Corn cob is an example of a natural material that can be considered as an agricultural waste. The objective of the present work is to improve the economic feasibility of corn cob by converting the original corn cob material into powder to be utilized in reinforcing epoxy-based composites. In the experiment, the corn cob was crushed and ground using a grain miller before it was characterized by scanning electron microscopy (SEM). The corn cob powder was added to the epoxy with different weight fractions (2, 4, 6, 8, 10 wt%). In order to prevent corn cob powder agglomeration and ensure homogeneous distribution of the reinforcement inside the epoxy, the ultrasonic technique and a mechanical stirrer were used. Then, the composite’s chemical compositions were evaluated using X-ray diffraction (XRD). The mechanical experiments showed an improvement in the Young’s modulus and compressive yield strength of the epoxy composites, increasing corn cob up to 8 wt% by 21.26% and 22.22%, respectively. Furthermore, tribological tests revealed that reinforcing epoxy with 8 wt% corn cob can decrease the coefficient of friction by 35% and increase wear resistance by 4.8%. A finite element model for the frictional process was constructed to identify different contact stresses and evaluate the load-carrying capacity of the epoxy composites. The finite element model showed agreement with the experimental results. An epoxy containing 8 wt% corn cob demonstrated the optimal mechanical and tribological properties. The rubbed surfaces were investigated by SEM to identify the wear mechanism of different composites.

## 1. Introduction

Recent global challenges and improvements associated with the utilization of different materials in tribological applications and polymer composites’ performance have attracted the attention of many researchers. Polymer composites are lightweight materials used in various applications such as automotive, satellites, marine, aerospace, etc. [1]. Furthermore, polymer composites have many advantages compared with metal materials, including low processing costs, corrosion resistance, lower raw material, and are non-toxic, which allow tremendous design flexibility [2]. Generally, polymer composites have been utilized in different applications and have shown outstanding results when used as a structural material for aerospace components [3]. These results motivated researchers to further investigate the tribological characteristics of polymer composites. Consequently, the tribological properties, including wear rate and friction coefficient of different polymer composites, have been studied to be used in nuts, brakes, bolts, bearing, and clutches [4,5,6]. To investigate the tribological characteristics, three essential matters should be studied [7]: the interfacial bonds strength; shear stress, which results in the delamination of surface contact area; and the contact area to illustrate the wear mechanism. Furthermore, additional factors can affect the composite performance during friction, such as the applied normal load and sliding velocity.

One of the most popular polymers used in different engineering applications is the epoxy thermoset, which has many advantages that encourage industrialists to use it. Epoxy-based thermosetting resin can be used for its chemical and heat resistance [8], high corrosion barrier [9], and it is an electric isolator, in addition to its high stiffness and the simplicity of its production and processing. Such advantages have nominated epoxy for various applications such as helicopter rotors, industrial flooring, pump impellers, and aircraft engines [10]. Nevertheless, epoxy has its drawbacks, such as its brittle nature, which affects its mechanical characteristics and decreases its wear resistance, preventing its utilization in frictional applications [11,12].

To enhance the properties of epoxy, researchers have tried to add some additives as a reinforcement and investigate the different characteristics of epoxy composites, including mechanical, tribological, electrical, and thermal qualities [13]. The mechanical and thermal properties of epoxy composites after adding block-copolymer (BCP) and core-shell rubber were investigated by Bajpai et al. [14]. They recorded an enhancement in the fracture toughness by 268% for 10 wt% BCP and 200% for 12 wt% CSR. Furthermore, adding 3 wt% BCP and CSR improved the fracture toughness by 100%. Kim et al. [15] investigated the effect of adding different weight fractions of graphene nanoplatelets on the mechanical properties of epoxy thermosets. They found that 4 wt% graphene nanoplatelets enhanced the fracture toughness and shear strength by 142% and 252%, respectively. Upadhyay et al. [16] sought to improve epoxy’s mechanical and tribological behavior by adding different weight fractions of nano-graphene. They found that increasing the graphene weight fraction up to 5 wt% enhanced the compressive stress. Furthermore, the specific wear rate decreased by approximately 50%, and the decrease in the friction coefficient reached 60%. Xian et al. [17] investigated the tribological performance of epoxy incorporated with different volume fractions of TiO_2_. They found that 4 vol% TiO_2_ could considerably decrease the specific wear rate. Utilizing the same additives with varying weight fractions, TiO_2_, Srivastava et al. [18] evaluated epoxy’s tribological and mechanical performance. The results recorded an enhancement in the epoxy composite wear resistance at 5 wt% in addition to an improvement in both impact strength and toughness. Bazrgari et al. [19] investigated the effect of incorporating Al_2_O_3_ with different weight fractions into epoxy matrix. They could prove that 1 wt% Al_2_O_3_ can improve the mechanical and tribological characteristics of the composite. Still, with the increase in Al_2_O_3_ weight fraction up to 3%, all the composite properties have deteriorated due to the additives’ agglomeration inside the matrix. Fouly et al. [20] studied the effect of reinforcing epoxy resin with a low loading fraction of Al_2_O_3_. They found that a 0.4 wt% Al_2_O_3_ could improve the epoxy composite’s mechanical and tribological properties.

The reinforcements used with different composites can be classified as natural and synthetic additives [1]. Synthetic additives are usually nonbiodegradable, and due to environmental pollution, the utilization of natural additives has attracted the attention of many researchers. Natural additives are easily available in nature at a low cost, are easily manufactured, and their utilization is eco-friendly [21]. Venkateshwaran et al. [22] evaluated the effect of reinforcing epoxy resin with banana fibers, and they recorded enhancement in tensile strength, flexural strength, and impact strength. Oladele et al. [23] investigated the effect of reinforcing epoxy resin with different weight fractions of eggshell powder. They recorded an enhancement in the tensile modulus, impact resistance, flexural properties, hardness, and wear resistance. Karthick et al. [24] reinforced PMMA resin with different weight fractions of seashell nanopowder and investigated its effect on the tribological properties of the polymer composite. They found that the optimal weight fraction that could enhance the tribological properties of the PMMA composite is 12 wt% seashell powder. Sim et al. [25] investigated the effect of reinforcing epoxy by fly ash on the mechanical properties of epoxy composites. Although the tensile strength was improved to a specific fly ash weight fraction, the compression strength was continuously enhanced as the fly ash weight fraction increased. Other researchers evaluated epoxy’s mechanical and wettability characteristics when reinforced by natural fibers [26].

Corn cob is considered one of the most common agricultural wastes, and some researchers have tried to turn it into a useful material. Chen et al. [27] extracted corn cob fibers and reinforced polyethylene. They found that the polyethylene composites recorded sufficient mechanical and weathering performance. Zhu et al. [28] reinforced high-density polyethylene (HDPE) by corn cob powder and investigated the composite water absorption and mechanical properties. The results showed that increasing corncob content led to an increase in the water absorption of the composite. The flexural strengths and moduli were increased up to 40% with increasing corn cob powder, but the mechanical properties started to deteriorate at a high level of powder content.

According to the above literature survey, using natural materials as a reinforcement could affect polymer composites’ mechanical and tribological properties. Although many researchers investigated the effect of corn cob as a reinforcement additive with polymers, there is a lack of research on the impact of a corn cob on the tribological properties of polymer composites. Consequently, the objective of the present investigation is to evaluate the performance of epoxy composite after incorporating corn cob powder with different weight fractions, 2, 4, 6, 8, and 10 wt%. The density, hardness, Young’s modulus, and compressive yield strength were determined based on the corn cob content. The tribological characteristics are introduced by evaluating the friction coefficient and specific wear rate of different composites. A finite element model was constructed based on the mechanical and tribological results to assess the load-carrying capacity. The wear mechanisms of each composite were identified after evaluating the worn surfaces using scanning electron microscopy (SEM).

## 2. Materials and Methods 

### 2.1. Preparation of Corn Cob Powder and Epoxy/Corn Cob Composite Samples

The matrix material utilized in the current study was the commercial-grade Kemapoxy 150 (CMB International, Wadi El Natroun City, Egypt) epoxy resin kit consisting of crystal-clear resin, a density of 1.11 ± 0.02 kg/L crystal-clear epoxy hardener. As mentioned before, corn cob is considered one of the most common agricultural wastes. Corn cob is the backbone part of the corn that the kernels grow around. After removing the kernels, corn cobs were dried under the sun for three months to decrease the moisture. Then, the corn cobs were initially crushed into smaller particles utilizing hammering before transferring the crushed powder to the mono chamber of a grain miller at a speed of 250 rpm for 3 h, with 15 min grinding and 15 min rest. Finally, the achieved powder was dried again in an oven at a temperature of 70 °C for 5 h to ensure the removal of all moisture.

**Figure 1:**
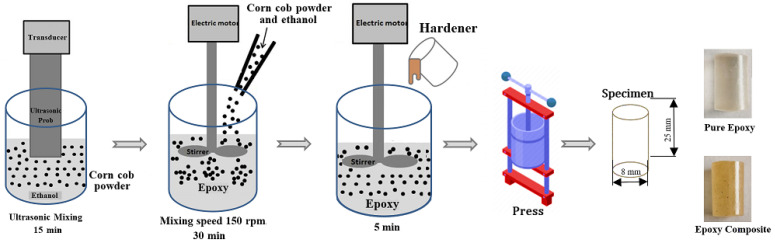
Schematic diagram of the epoxy composite samples’ fabrication.

The fabrication of tested samples passed through different stages. First, the corn cob powder was added to ethanol and mixed ultrasonically for 15 min to prevent powder agglomeration. Second, the liquid epoxy was added. To ensure homogenous dispersion of the corn cob powder inside the liquid epoxy, mixing using a mechanical stirrer was applied to blend the whole mixture for 30 min at 150 rpm. Thirdly, the epoxy/ethanol/corn cob mixture was inserted into a vacuum chamber to remove any voids and to fumigate the ethanol for 24 h. Fourthly, the hardener was added to the mix in a ratio of 1:2 to the epoxy weight and mixed again for 5 min. Eventually, the mixture was discharged into a mold taking the shape of needed specimens and placed again in a vacuum at 35 ◦C. A schematic diagram illustrating the fabrication process is shown in Figure 1. Based on the epoxy supplier instructions, the fabricated samples were left for 7 days before applying any test to achieve the recommended mechanical characteristics. The epoxy/corn cob composites were prepared by adding different weight fractions of corn cob. Table 1 illustrates the weight percentage of each material and the code of each sample.

### 2.2. Mechanical Properties of Epoxy/Corn Cob Composites

The mechanical characteristics of the fabricated epoxy/corn cob composites were evaluated to investigate the effect of adding corncob powder with different weight fractions. According to ASTM D2240 [29], the D-index’s shore hardness was evaluated for each composite sample. The utilized durometer specifications were 15 s dwell time with a 5 ± 0.5 kg loading. To ensure the hardness of the produced composite, measuring the hardness was conducted on different regions along the surfaces of the specimens, and the average value and standard errors were calculated. According to ISO 604:2002 Plastics [30], to apply the compression test, epoxy/corn cob samples were fabricated with a length and diameter of 16 mm and 8 mm, respectively. A 30-ton computer-controlled universal testing machine was utilized to apply the compression test with a 2 mm/min strain rate. Based on the compression test results, the elastic modulus and compressive yield strength were evaluated.

### 2.3. Tribological Properties of Epoxy/Corn Cob Composites

**Figure 2:**
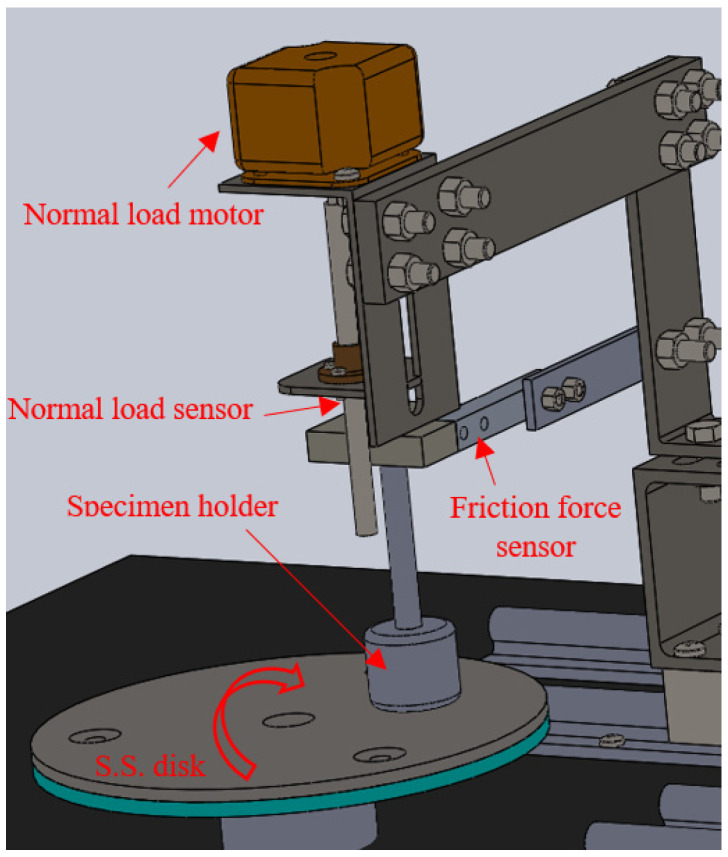
Pin-on-disk tribometer.

The tribological performance of epoxy/corn cob composites was evaluated in dry conditions according to ASTM G99-95 [31]. A pin-on-disc tribometer was utilized at room temperature 24:27 °C and humidity around 60%, as shown in Figure 2. The epoxy/corn cob sample was considered the tribometer pin with a cylindrical shape with a diameter and length of 8 mm and 25 mm, respectively. The composite samples were rubbed against a stainless-steel disk with a diameter of 18 cm and surface roughness of 12.5 μm. To prevent errors in the tribological results, the stainless-steel disk was cleaned and dried before every experiment to remove any remaining contaminants. The epoxy composite tribological performance was evaluated under normal loads, and sliding distances changed while the sliding speed was constant at 0.4 m/s. For each load, sliding distance, and composite composition, the friction test was repeated 6 times before calculating the specific wear rate and friction coefficient. The specific wear rate (*SWR*) was calculated using Equation (1), dependent on sliding distances (*L*), the difference in weight before and after the test (Δ*m*), the density of each composite sample measured by Archimedes’ principle (*ρ*), and the applied normal load (*F_n_*).

(1) $$SWR=\frac{\Delta m}{\rho \;L\;F_{n}}$$

## 3. Results and Discussion

**Figure 3:**
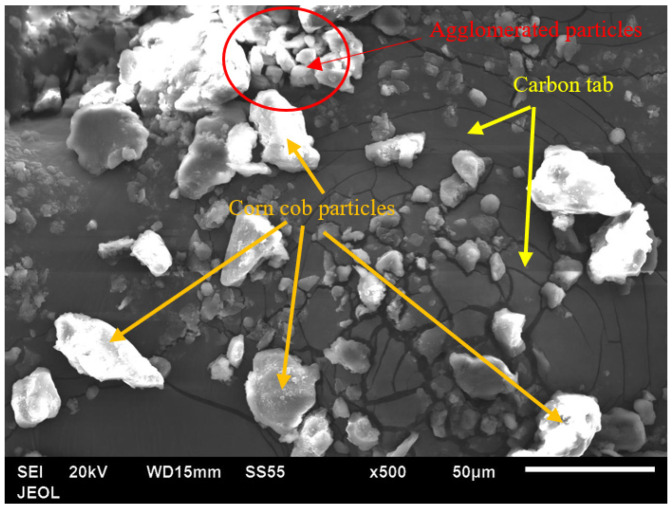
SEM image of corn cob particles after the grain milling process.

After the grain milling process, the corn cob particles’ morphology was characterized utilizing a scanning electron microscope (SEM). As shown in Figure 3, the produced corn cob powder had an irregular, rugged shape with a rough surface and some large particles covered with agglomerates. Furthermore, some particles have much smaller sizes and are agglomerated, as shown the red circle.

**Figure 4:**
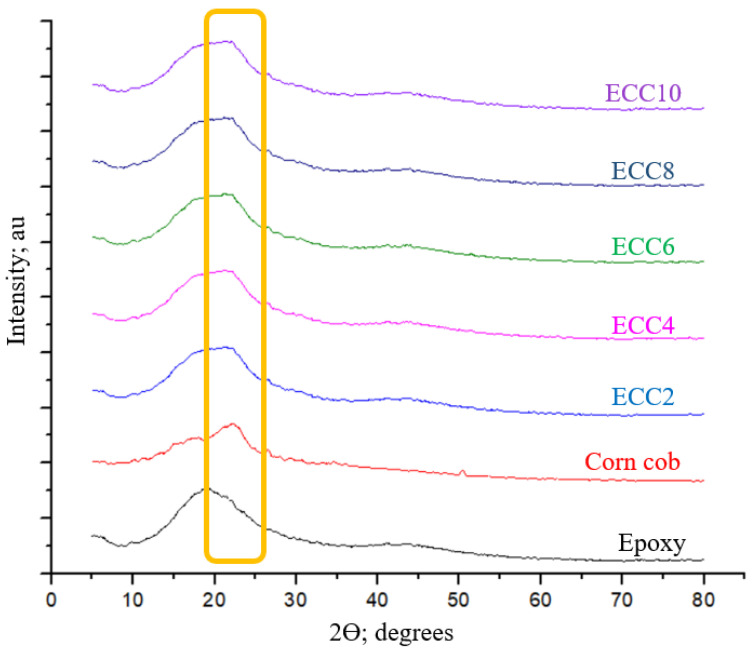
X-ray diffraction (XRD) traces of neat epoxy, corn cob powder, and epoxy composites.

To investigate the effect of incorporating corn cob powder into epoxy resin on the chemical composition of the composites, X-ray diffraction (XRD) was utilized. Figure 4 shows that the carbon peak is the major peak of pure epoxy, and it obviously appears in all epoxy composites with different weight fractions of corn cob. In addition, the XRD shows that the main peak of epoxy is not sharp, which illustrates its amorphous nature [32]. There is also one major peak of corn cob powder at 21.8°, similar to the XRD pattern of alpha cellulose [33]. Incorporating corn cob powder in epoxy resin affected the main peak of epoxy, as shown in the yellow rectangle in Figure 3. However, the disappearance of new peaks indicates that corn cob incorporation did not affect the epoxy structural characteristics, and no chemical reaction occurred between the epoxy and corn cob. The increasing weight fraction of corn cob did not affect the amorphous nature of the epoxy, and the appearance of the corn cob peak with the same effect in all composites emphasizes its thorough distribution inside the epoxy resin.

**Figure 5:**
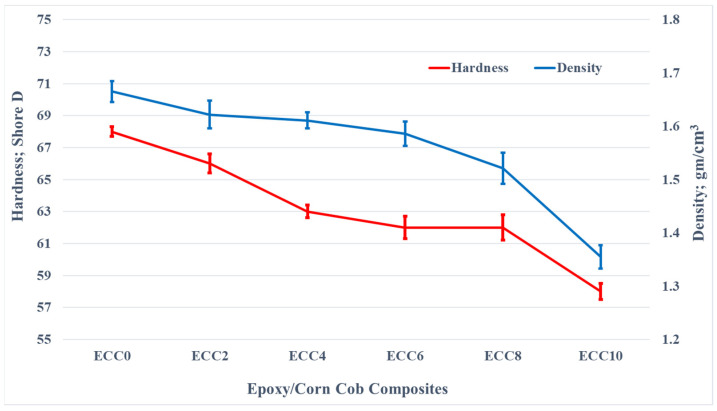
Density and shore-D hardness of epoxy/corn cob composites.

The density of the produced composite is one of the most important properties that can affect the weight of the new generated composite. The density of composite materials usually depends on the density of both the matrix and reinforcement materials. Consequently, the current study used Archimedes’ principle to calculate the density of the fabricated epoxy/corn cob composites. Figure 5 illustrates the variation in the epoxy composite density due to the corn cob weight fraction change. The density was found to be reduced as the corn cob weight fraction increased. Increasing the corn cob content to 10 wt% of the epoxy matrix decreased the composite density by 22.87%. This decrease in density can lead to a lighter polymer and support the advantages of polymer utilization in different applications. The reduction in the epoxy composite could be attributed to replacing wt% of the epoxy by a wt% of a lower density material, corn cob.

In the same figure, Figure 5, the effect of incorporating corn cob on the epoxy composites hardness is illustrated. It was evident that increasing corn cob weight fraction led to a decrease in the hardness of the composites. The average shore-D hardness of pure epoxy was 68. Increasing the corn cob up to 10 wt% decreased the hardness by 17.24% to reach 58 on the D-index scale. Due to the increased weight fraction of the corn cob, the density of the composite samples decreased; as a result, a reduction in hardness might occur [34].

**Figure 6:**
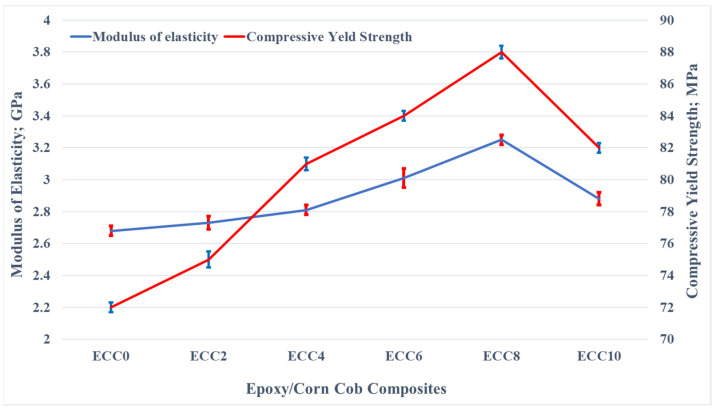
Young’s modulus and compressive yield strength of epoxy/corn cob composites.

The experimental results of the compression test of epoxy corn cob composites are shown in Figure 6. It was noticed that increasing corn cob content contributed to the improvement of the epoxy composites’ Young’s modulus along with the compressive yield strength. The epoxy composite consisting of 8 wt% corn cob was the optimum one concerning the mechanical strength. Young’s modulus and compressive yield strength enhancement reached 21.26% and 22.22%, respectively. The improvement in the compressive yield strength might be due to the thorough distribution of corn cob particles inside the epoxy matrix that helped the matrix transfer the compressive load to the corn cob powder, which dissipated it. 

On the other hand, incorporating 10 wt% corn cob resulted in the deterioration of Young’s modulus and the compressive yield strength. This result could be attributed to the ease of debonding corn cob particles from the epoxy matrix because of the high corn cob weight fraction. Consequently, the load transmission from the epoxy to the corn cob particles failed; hence the mechanical characteristics diminished.

**Figure 7:**
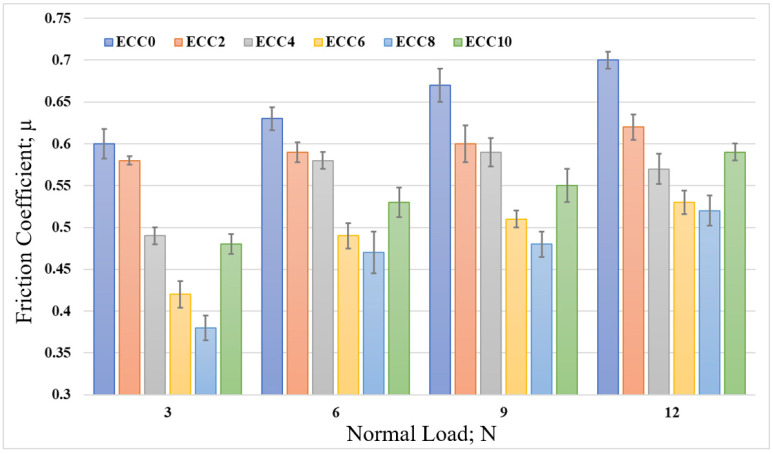
The friction coefficient of epoxy/corn cob composites under different normal loads.

The variation in the friction coefficient of neat epoxy and 2–10 wt% corn cob particles-reinforced composite under different normal loads (3–12 N) is illustrated in Figure 7. Figure 7 shows that the incorporation of corn cob particles in the epoxy resin decreased the coefficient of friction with the variation in the normal loads. The results show that 8 wt% corn cob recorded the minimum friction coefficient (0.39), 35% less than that for pure epoxy (0.6) at 3 N normal load. However, adding more particles of corn cob to reach 10 wt% increased the friction coefficient. More analysis is offered later in the current study to understand why the friction coefficient increased when corn cob weight fraction reached 10 wt%. On the other hand, Figure 7 showed that increasing the normal load led to an increase in the friction coefficient. This might have happened because of the thermal effect caused by the high normal load [35], in which the contact area may be changed, leading to a change in the adhesion between the tested specimens and the stainless steel disk. Chang et al. [36] proved that increasing the contact temperature increases the friction coefficient.

**Figure 8:**
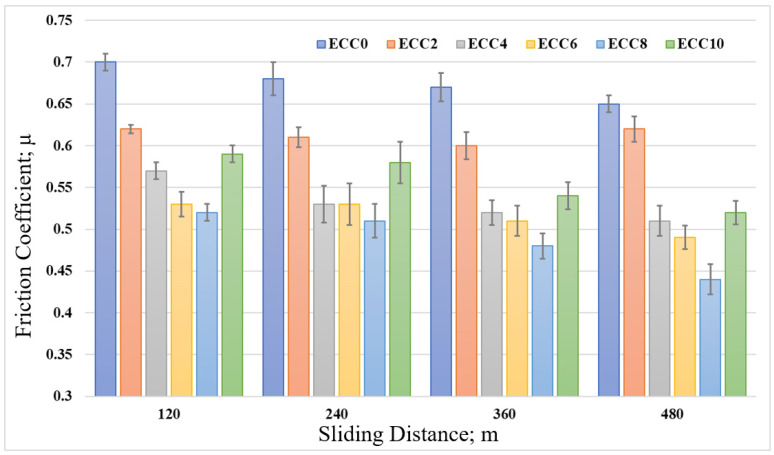
Friction coefficient of epoxy/corn cob composites with different sliding distances.

**Figure 9:**
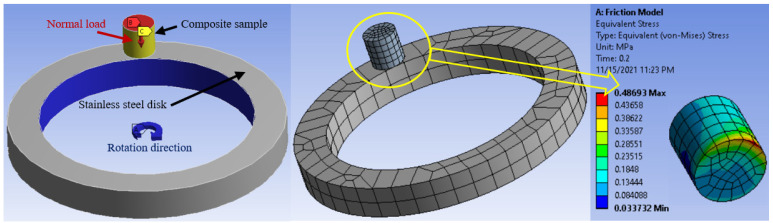
Finite element model of the frictional process with boundary condition, meshing, and composite sample after friction.

Figure 8 shows the influence of the sliding distance on the friction coefficient of epoxy/corn cob composites. The sliding distances were varied from 120 m to 480 m under a normal load of 12 N. Generally, the friction coefficient decreased with the increase in the sliding distance, while 8 wt% corn cob still recorded the lowest coefficient of friction among other composites. The decrease in the friction coefficient could be attributed to the smoothness in the epoxy composite surface after rubbing for a long distance against the stainless steel disk. Furthermore, Dass et al. [10] reported that after rubbing polymeric samples for a long time, the generated heat could melt the surface of the composite. Consequently, a thin film of the melted polymer composite is transmitted to the counterpart. It works as a lubrication layer between the rubbed surfaces, resulting in a decrease in the friction coefficient. To conclude, the reduction in the epoxy/corn cob coefficient of friction stands for an enhancement in the epoxy composite load-carrying capacity due to the existence of corn cob particles. Kuminek et al. [37] claimed that the contact stress on the surface of rubbed samples could indicate the load-carrying capacity. Therefore, in the current study, the pin on the disk tribometer was modeled utilizing ANSYS software, as shown in Figure 9, and the stresses due to the frictional process were reported.

Figure 9 illustrates the finite element model for the frictional process. The disk was modeled as a hollow cylinder with a stainless steel specification, and the epoxy composite was modeled as a pin. The measured mechanical properties of composite samples and the measured friction coefficient between the composite samples and the stainless-steel disk were inserted into the ANSYS software. In order to measure different stresses during the rubbing process, frictional contact was defined between the pin and the disk. Normal Lagrange contact solution is commonly used when normal pressure is applied in the z-direction. In addition, output results are dependent on samples stiffness. In the present work, the normal force was solved explicitly and was added as an additional degree of freedom applied in the z-direction with 12 N. The automatic meshing provided by ANSYS software was utilized both the pin and the disk, as shown in the middle of Figure 9. After the automatic meshing, the generated grids for both the pin and the disk were a combination of tetrahedron and hexahedron shapes. The disk’s elements and nodes are 214 and 1623, respectively. The pin’s elements and nodes are 112 and 637, respectively. The boundary conditions for the epoxy composite pin were a fixation in the x and y direction besides a 12 N normal load on the sample surface at the z-direction. A 300 rpm speed was applied to the rotational joint of the stainless steel disk. The stress on the surface of the epoxy composite surfaces after the frictional process is illustrated in Figure 9. It was evident that the motion direction generates concentrated stress on the sample edge, where the deterioration of the sample surface may begin.

**Figure 10:**
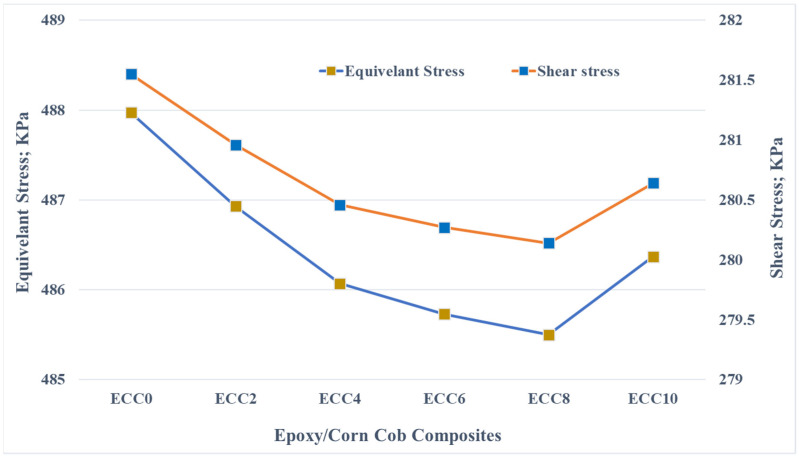
Contact stresses on the surface of the epoxy/corn cob composites.

The finite element results show the generated equivalent and shear stresses variation due to the friction between different epoxy composite samples and the stainless steel disk, as shown in Figure 10. It was evident that the finite element results were affected by the mechanical properties of the fabricated composites. As the corn cob content increased, an enhancement in the samples strength was recorded, which is reflected in the finite element results as a decrease in both the equivalent and shear stresses. This decrease in the contact stresses corresponds to an improvement in the load-carrying capacity that caused a reduction in the friction coefficient [38]. Marra et al. [39] claimed that the decrease in the shear stress between contact elements could reduce the friction coefficient.

**Figure 11:**
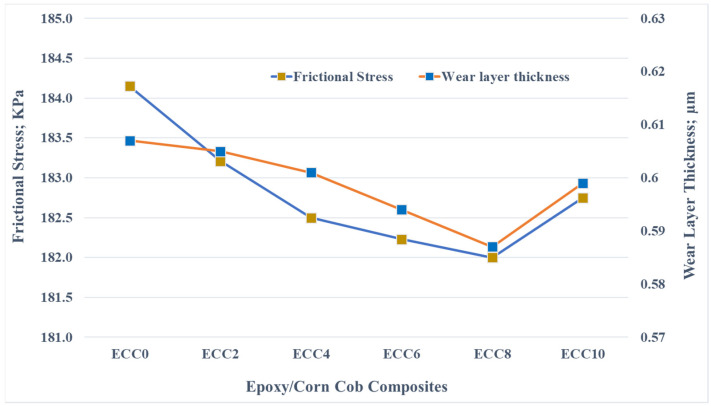
Frictional stress and wear layer thickness on the surface of the epoxy/corn cob composites.

Figure 11, containing finite element results, shows that the change in frictional stress generated during the rubbing process is similar to the equivalent and shear stress changes. The contact stresses can be the main reason for the deterioration of the composite samples. Therefore, wear layer thickness was recorded during the simulation of the frictional process. The results show that the wear layer thickness decreases with corn cob weight fraction. These results could also be attributed to the enhancement in the composite strength recorded during the evaluation of the mechanical characterization.

**Figure 12:**
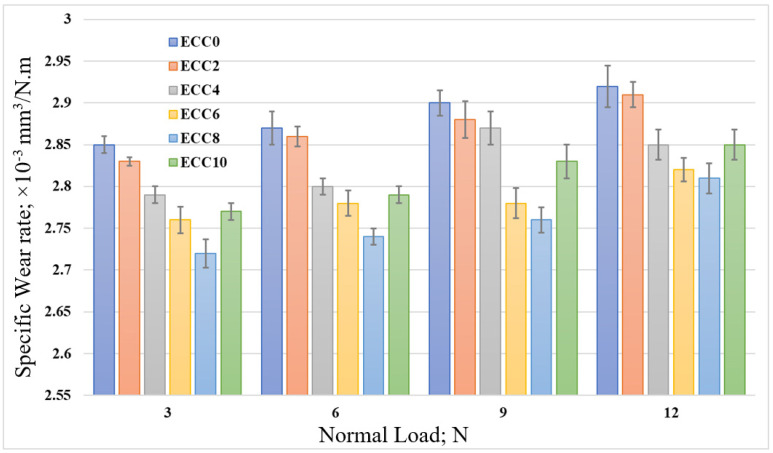
The specific wear rate of epoxy/corn cob composites under different normal loads.

**Figure 13:**
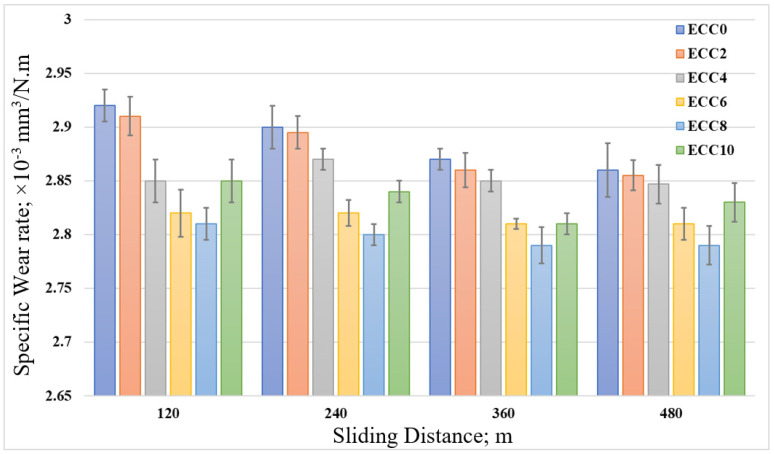
The specific wear rate of epoxy/corn cob composites with different sliding distances.

To validate the finite element results and evaluate the influence of different corn cob weight fractions on the tribological performance of epoxy composites, the specific wear rate of the epoxy composites under different normal loads was calculated. Figure 12 shows that incorporating corn cob particles in the epoxy resin decreased the specific wear rate. According to the results, 8 wt% corn cob recorded the minimum specific wear rate. Nevertheless, the extra addition of corn cob to reach 10 wt% increased the specific wear rate. The increase in wear rate to more than 8 wt% corn cob could be attributed to the agglomeration of corn cob particles due to high weight percent [25]. Furthermore, the high reinforcement weight ratio and the poor interfacial bonding between the epoxy and the corn cob particles resulted in a decrease in the wear resistance of the epoxy composite. Thus, results indicate an agreement among the finite element results, wear layer thickness, and the experimental results with the specific wear rate. On the other hand, increasing the normal load led to an increase in the specific wear rate. During the rubbing process, some abrasive particles detached from the surface of the composite samples. With high loading, these particles were pushed against the sample surface and increased the frictional force. The increase in the frictional force may deteriorate the sample’s surface, and, consequently, increase the specific wear rate. Figure 13 illustrates the change in the specific wear rate of epoxy/corn cob composites after sliding for different distances. A limited gradual decrease in the specific wear rate was recorded for all epoxy composites.

**Figure 14:**
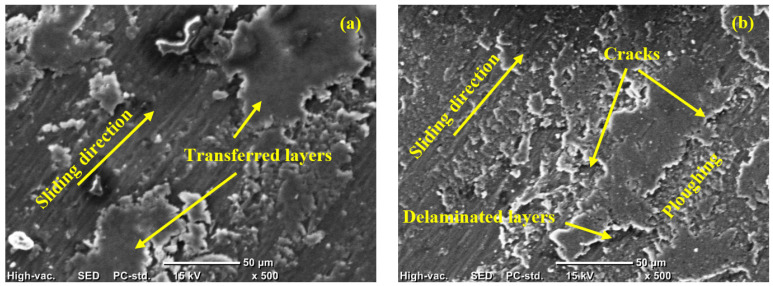

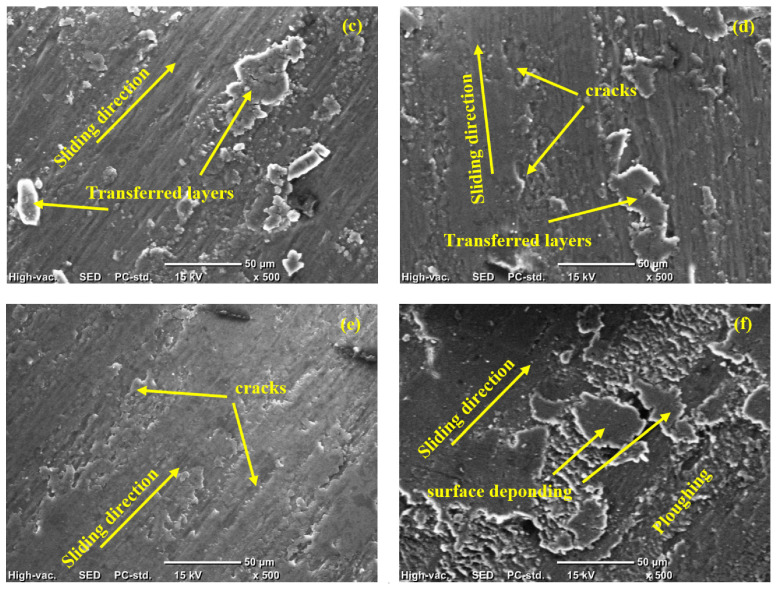
SEM of worn epoxy composite surfaces (**a**) ECC0, (**b**) ECC2, (**c**) ECC4, (**d**) ECC6, (**e**) ECC8, and (**f**) ECC10.

The microscopic analysis of epoxy composites rubbed surfaces were utilized to identify the wear mechanism during the friction process, as shown in Figure 14. The high shear stress besides the plowing action resulted in a degradation of the rubbed surface of the pure epoxy. Therefore, Figure 14a shows some eliminated layers and severe insensitive damage on the pure epoxy surface. The illustrated deterioration in the epoxy surface explains the cause of the increased specific wear rate. Furthermore, the weak separated layers increased the shear resistance; thus, the measured friction coefficient increased [40]. The rubbed surface depicts diversified wear mechanisms such as micro-cutting; its propagation resulted in the debonding of some material from the epoxy sample surface. Therefore, the primary wear mechanism could be a delamination mechanism, which usually encourages the increase in both specific wear mechanism and the friction coefficient [41]. Incorporating 2 wt% corn cob into the epoxy matrix enhanced the epoxy composite’s thrust-bearing capacity, leading to fewer eliminated layers with a clear plowing surface and the initiation of microcracks, as shown in Figure 14b. However, as the corn cob content is low, still eliminated layers of the epoxy appeared on the surface of the rubbed sample. Compared to pure epoxy, adding 2 wt% corn cob enhanced the strength of the composite surface and decreased the specific wear rate and the change in the wear mechanism to be a fatigue-delamination due to the appearance of the micro cracks. The addition of 4 wt% corn cob to the epoxy matrix led to more enhancement and the eliminated layers decreased again, as shown in Figure 14c. In the case of 6 wt% corn cob, Figure 14d, the eliminated layers retracted again, indicating an enhancement in the composite sample’s strength. The rubbed surface becomes smoother with micro-plowing, as a wear mechanism, and the clear view on the surface is the appearance of the microcracks. Figure 14e illustrates the worn surface of the epoxy composite after adding 8 wt% corn cob. The delaminated layers disappeared, and only some micro-cracks appeared on the surface of the composite sample. The increase in the presence of the corn cob provided improved strength to resist the generated thrust force on the epoxy composite surface during the friction process. Consequently, an enhancement in the wear resistance was achieved that indicates perfect interfacial adhesion between the corn cob particles and the epoxy, which encourages the epoxy matrix to share generated thrust force and shear stress on the sample surface. Therefore, an enhancement in the friction coefficient and specific wear rate was achieved for 8 wt%. Lastly, Figure 14f shows the worn surface of epoxy composite with 10 wt% corn cob. As mentioned before, studies proved that the high reinforcement weight ratio and the poor interfacial bonding among the composite composition reduce the capacity of the corn cob particles to support the epoxy surface against applied stress. Consequently, the wear resistance decreased, resulting in a surface debonding and severe damage. Furthermore, separated layers and particles increased plowing action, and shear stress increased the shear resistance; thus, the measured friction coefficient increased.

## 4. Conclusions

In conclusion, we conducted an experimental investigation on reinforcing epoxy-based polymer using corn cob, a natural material. The physical, mechanical, and tribological characteristics of the fabricated epoxy/corn cob composites were evaluated at different corn cob weight fractions, and the following conclusions are drawn:

- The results revealed that corn cob could be used as a natural reinforcement for polymer-based composites.
- Without using an agent to enhance the bonding between the epoxy and the corn cob, the weight fraction of the corn cob is limited to 8 wt%.
- Increasing the corn cob weight fraction to 10 wt% decreased both density and hardness of the composite by 22.87% and 17.24%, respectively.
- For 8 wt% corn cob, Young’s modulus and compressive yield strength of the epoxy composite reached 21.26% and 22.22%, respectively.
- For 8 wt% epoxy/corn cob, the composite recorded a frictional coefficient of 35% less than pure epoxy, as well as enhanced wear resistance.
- The finite element results showed an enhanced load-carrying capacity due to corn cob incorporation.

Finally, corn cob particles could be modified chemically to improve the interfacial bond between the epoxy and corn cob particles and to reinvestigate the new composite characteristics.

## Figures and Tables

**Table 1:** Composition of epoxy/corn cob composite samples.

Code	Corn Cob wt%	Epoxy wt%	Hardener wt%
ECC0	0	66.67	33.33
ECC2	2	65.33	32.67
ECC4	4	64	32
ECC6	6	62.67	31.33
ECC8	8	61.33	30.67
ECC10	10	60	30

## Data Availability

Not applicable.

## References

[1] Chavali P.J., Taru G.B. (2021). Effect of Fiber Orientation on Mechanical and Tribological Properties of Banana-Reinforced Composites. J. Fail. Anal. Prev..

[2] Jaramillo A., Medina C., Flores P., Canales C., Maldonado C., Rivera P.C., Rojas D., Meléndrez M., Castaño P. (2020). Improvement of thermomechanical properties of composite based on hydroxyapatite functionalized with alkylsilanes in epoxy matrix. Ceram. Int..

[3] Fouly A., Ibrahim A.M.M., Sherif E.-S.M., FathEl-Bab A.M.R., Badran A.H. (2021). Effect of Low Hydroxyapatite Loading Fraction on the Mechanical and Tribological Characteristics of Poly (Methyl Methacrylate) Nanocomposites for Dentures. Polymers.

[4] Yousif B.F. (2013). Design of newly fabricated tribological machine for wear and frictional experiments under dry/wet condition. Mater. Des..

[5] Bajpai P.K., Singh I., Madaan J. (2013). Tribological behavior of natural fiber reinforced PLA composites. Wear.

[6] Ibrahim A.M.M., Mohamed A.F.A., Fathelbab A.M., Essa F.A. (2018). Enhancing the tribological performance of epoxy composites utilizing carbon nano fibers additives for journal bearings. Mater. Res. Express.

[7] Farfan-Cabrera L.I., Tapia-Gaspar M., Pérez-González J. (2021). Tribology of Polymer Matrix Composites within the Automotive Industry.

[8] Kim K., Kim M., Hwang Y., Kim J. (2014). Chemically modified boron nitride-epoxy terminated dimethylsiloxane composite for improving the thermal conductivity. Ceram. Int..

[9] Rusly SN A., Ismail I., Matori K.A., Abbas Z., Shaari A.H., Awang Z., Ibrahim I.R., Idris F.M., Zaid M.H.M., Mahmood M.K.A. (2020). Influence of different BFO filler content on microwave absorption performances in BiFeO3/epoxy resin composites. Ceram. Int..

[10] Dass K., Chauhan S.R., Gaur B.V. (2017). Study on the effects of nano-aluminum-oxide particulates on mechanical and tribological characteristics of chopped carbon fiber reinforced epoxy composites. Proc. Inst. Mech. Eng. Part J. Mater. Des. Appl..

[11] Che Y., Sun Z., Zhan R., Wang S., Zhou S., Huang J. (2018). Effects of graphene oxide sheets-zirconia spheres nanohybrids on mechanical, thermal and tribological performances of epoxy composites. Ceram. Int..

[12] Fouly A., Ibrahim A.M.M., El-Bab A.M. (2020). Promoting the tribological Properties of epoxy Composites via using Graphene Nanoplatelets as a functional Additive. KGK-Kautsch Gummi Kunstst..

[13] Mostovoy A., Yakovlev A., Tseluikin V., Lopukhova M. (2020). Epoxy nanocomposites reinforced with functionalized carbon nanotubes. Polymers.

[14] Bajpai A., Wetzel B., Klingler A., Friedrich K. (2020). Mechanical properties and fracture behavior of high-performance epoxy nanocomposites modified with block polymer and core–shell rubber particles. J. Appl. Polym. Sci..

[15] Kim J., Cha J., Chung B., Ryu S., Hong S.H. (2020). Fabrication and mechanical properties of carbon fiber/epoxy nanocomposites containing high loadings of noncovalently functionalized graphene nanoplatelets. Compos. Sci. Technol..

[16] Upadhyay R.K., Kumar A. (2019). Effect of particle weight concentration on the lubrication properties of graphene based epoxy composites. Colloid Interface Sci. Commun..

[17] Xian G., Walter R., Haupert F. (2006). Friction and wear of epoxy/TiO2 nanocomposites: Influence of additional short carbon fibers, Aramid and PTFE particles. Compos. Sci. Technol..

[18] Srivastava S., Tiwari R.K. (2012). Synthesis of epoxy-TiO2 nanocomposites: A study on sliding wear behavior, thermal and mechanical properties. Int. J. Polym. Mater..

[19] Bazrgari D., Moztarzadeh F., Sabbagh-Alvani A.A., Rasoulianboroujeni M., Tahriri M., Tayebi L. (2018). Mechanical properties and tribological performance of epoxy/Al2O3 nanocomposite. Ceram. Int..

[20] Fouly A., Alkalla M.G. (2020). Effect of low nanosized alumina loading fraction on the physicomechanical and tribological behavior of epoxy. Tribol. Int..

[21] Daramola O.O., Akinwekomi A.D., Adediran A.A., Akindote-White O., Sadiku E.R. (2019). Mechanical performance and water uptake behaviour of treated bamboo fibre-reinforced high-density polyethylene composites. Heliyon.

[22] Venkateshwaran N., ElayaPerumal A., Alavudeen A., Thiruchitrambalam M. (2011). Mechanical and water absorption behaviour of banana/sisal reinforced hybrid composites. Mater. Des..

[23] Oladele I.O., Makinde-Isola B.A., Adediran A.A., Oladejo M.O., Owa A.F., Olayanju T.M.A. (2020). Mechanical and wear behaviour of pulverised poultry eggshell/sisal fiber hybrid reinforced epoxy composites. Mater. Res. Express.

[24] Karthick R., Sirisha P., Sankar M.R. (2014). Mechanical and tribological properties of PMMA-sea shell based biocomposite for dental application. Procedia Mater. Sci..

[25] Sim J., Kang Y., Kim B.J., Park Y.H., Lee Y.C. (2020). Preparation of fly ash/epoxy composites and its effects on mechanical properties. Polymers.

[26] Atmakuri A., Palevicius A., Siddabathula M., Vilkauskas A., Janusas G. (2020). Analysis of mechanical and wettability properties of natural fiber-reinforced epoxy hybrid composites. Polymers.

[27] Chen B., Cai D., Luo Z., Chen C., Zhang C., Qin P., Cao H., Tan T. (2018). Corncob residual reinforced polyethylene composites considering the biorefinery process and the enhancement of performance. J. Clean. Prod..

[28] Zhu S., Guo Y., Tu D., Chen Y., Liu S., Li W., Wang L. (2018). Water absorption, mechanical, and crystallization properties of high-density polyethylene filled with corncob powder. BioResources.

[29] Zhao H., Allanson D., Ren X.J. (2015). Use of shore hardness tests for in-process properties estimation/monitoring of silicone rubbers. J. Mater. Sci. Chem. Eng..

[30] BS EN ISO (1999). 14126: 1999-Fibre-Reinforced Plastic Composites. Determination of Compressive Properties in the In-Plane Direction.

[31] ASTM (2008). G99, Standard Test Method for Wear Testing with a Pin-on-Disk Apparatus.

[32] Sharmila T.B., Antony J.V., Jayakrishnan M.P., Beegum P.S., Thachil E.T. (2016). Mechanical, thermal and dielectric properties of hybrid composites of epoxy and reduced graphene oxide/iron oxide. Mater. Des..

[33] Yu J., Qian Q., Zhao X. (2009). Research progress on effects of structural planes of rock mass on stress wave propagation law. Acta Armamentarii.

[34] Guo R.Q., Rohatgi P.K., Nath D. (1997). Preparation of aluminium-fly ash particulate composite by powder metallurgy technique. J. Mater. Sci..

[35] Khun N.W., Zhang H., Lim L.H., Yue C.Y., Hu X., Yang J. (2014). Tribological properties of short carbon fibers reinforced epoxy composites. Friction.

[36] Chang L., Zhang Z., Zhang H., Friedrich K. (2005). Effect of nanoparticles on the tribological behaviour of short carbon fibre reinforced poly (etherimide) composites. Tribol. Int..

[37] Kuminek T., Aniołek K., Młyńczak J. (2015). A numerical analysis of the contact stress distribution and physical modelling of abrasive wear in the tram wheel-frog system. Wear.

[38] Tang W., Zhou Y., Zhu H., Yang H. (2013). The effect of surface texturing on reducing the friction and wear of steel under lubricated sliding contact. Appl. Surf. Sci..

[39] Marra K.G., Szem J.W., Kumta P.N., DiMilla P.A., Weiss L.E. (1999). In vitro analysis of biodegradable polymer blend/hydroxyapatite composites for bone tissue engineering. J. Biomed. Mater. Res. Off. J. Soc. Biomater. Jpn. Soc. Biomater. Aust. Soc. Biomater. Korean Soc. Biomater..

[40] Fouly A., Almotairy S.M., Aijaz M.O., Alharbi H.F., Abdo H.S. (2021). Balanced Mechanical and Tribological Performance of High-Frequency-Sintered Al-SiC Achieved via Innovative Milling Route—Experimental and Theoretical Study. Crystals.

[41] Ibrahim A.M.M., Shi X., Radwan A.R., Mohamed A.F.A., Ezzat M.F. (2019). Enhancing the tribological properties of NiAl based nano-composites for aerospace bearing applications. Mater. Res. Express.

